# Effect of L-arginine and sildenafil citrate on intrauterine growth restriction fetuses: a meta-analysis

**DOI:** 10.1186/s12884-016-1009-6

**Published:** 2016-08-16

**Authors:** Juncao Chen, Xiaoyuan Gong, Pingyang Chen, Kaiju Luo, Xiuquan Zhang

**Affiliations:** 1 Division of Neonatology, The Second Xiangya Hospital, Central South University, 139 Renmin Middle Rd, Changsha, Hunan 410011 China; 2 Department Obstetrics and Gynecology and Reproductive Genetics, University of Utah, Salt Lake City, UT 84132 USA

**Keywords:** L-arginine, Sildenafil citrate, Nitric oxide, Intrauterine growth restriction, Birth weight, Gestational age

## Abstract

**Background:**

Intrauterine growth restriction (IUGR) is associated with perinatal morbidity and mortality. Several clinical trials have reported L-arginine and sildenafil citrate had effect on intrauterine growth restriction fetuses. A meta-analysis of available randomized controlled trials (RCTs) was conducted to investigate the effects of L-arginine and sildenafil citrate on major clinical outcomes of IUGR fetuses.

**Methods:**

Systematically searched Medline, Embase, the Cochrane Library, and Clinical Trials, references of retrieved articles, and conference proceedings from 1960 to 2015. We included randomized controlled trials assessing the effects of L-arginine and sildenafil citrate on IUGR. Outcomes analyzed were the birth weight, gestational age at labor, Apgar score at 1and 5 min, the ratio of NRDS, the ratio of ICH and neonatal death, etc.

**Results:**

Ten trials were included. Nine trials (576 patients) compared L-arginine with either placebo or no intervention. In the L-arginine treatment groups of the L-arginine trials, there was a significant increase in fetal birth weight (SMD 0.41, 95 % CI [0.24,0.58]), gestational age (SMD 0.30, 95 % CI [0.07,0.54]); L-arginine treatment group have a significant reduction in the ratio of neonatal respiratory distress syndrome (*P* = 0.009), intracranial hemorrhage of fetuses (*P* = 0.002), but the number of included studies and people on these outcomes are small. As only one trial (41 patients) compared sildenafil citrate with placebo, it was too small for reliable conclusions about possible differential effects could be drawn.

**Conclusions:**

The results of this meta-analysis showed that L-arginine increased birth weight and prolonged gestational age at labor of IUGR fetuses. However, further large-scale RCTs are needed to adequately assess the effect of L-arginine and Sildenafil citrate on clinical outcomes, because the number of study may be small.

**Electronic supplementary material:**

The online version of this article (doi:10.1186/s12884-016-1009-6) contains supplementary material, which is available to authorized users.

## Background

Fetal development represents a critical period in humans, and can influences adult phenotypes. Intrauterine growth restriction (IUGR), a condition in which the fetus is pathologically growth restricted in uterus, remains a serious public health problem. Although the term is often used interchangeably with small for gestational age (SGA), which refers to for infants with birth weights below the 10th percentile for the gestational age. IUGR is estimated to occur in 5 to 7 % of all pregnancies [1]. The most common cause of IUGR is placenta-vascular insufficiency. Many studies have shown that IUGR is associated with increased risk of premature birth, reduced neonatal survival and long-term sequelae (including impaired neuro-developmental progress in childhood and insulin-resistance in adulthood) [2].

Nitric oxide (NO) is produced by nitric oxide synthases (NOS) using the amino acid L-arginine as substrate. Then NO diffuses into adjacent vascular smooth muscle cells and increases the concentration of second-messenger cyclic guanosine monophosphate (cGMP), resulting in the relaxation of vascular smooth muscle. In a normal pregnant woman, NO plays an important role in increasing the oxygen and nutrient supply to the fetus by influencing vasodilatation in feto-placental circulation. Reduced NO availability may have an important role in the pathophysiology of IUGR [3]; previous data suggest that IUGR could be induced by blocking NO synthesis [4]. Therefore, NO donors (glyceryl trinitrate and isosorbide mononitrate), precursors (L-arginine) and NO mediator (sildenafil citrate and vardenafil) may be possible therapeutic approaches for IUGR. In these NO agents, L-arginine or sildenafil citrate supplementation in pregnancy has recently been applied to manage IUGR and its complications in many clinical studies.

L-arginine is an amino acid that is essential to the body’s production of NO. However, many studies had shown that preterm infants and pregnant women could present with arginine deficiency [5, 6], and impaired arginine transport into endothelial cells was observed in the umbilical endothelium from IUGR infants [7]. Sildenafil citrate, a phosphodiesterase inhibitor (PDE5-selective inhibitor) that enhances the effects of NO, acts by blocking the enzymes that break down cGMP, which mediates the effects of NO in the body and leads to vascular relaxation.

Some studies had shown that the use of L-arginine and sildenafil citrate appeared effective. However, other clinical trials completed in the past few years presented uncertain results. Therefore, we carried out a systematic review and meta-analysis to investigate the effects of L-arginine and sildenafil citrate on clinical outcomes of IUGR.

## Methods

In October 2015 we searched Medline (from 1966 to October 2015), Embase (from 1974 to October 2015), the Cochrane Library database (Cochrane Central Register of Controlled Trials) and Clinical Trials (October 2015); The search terms that were used to searched original articles included “sildenafil citrate”, “L-arginine”,”arginine”, “intrauterine growth restriction”, “fetal growth restriction”, “small for gestational age”, and “low birth weight”. These search terms were combined according to the need of the study. Reference lists from identified trials and review articles also were manually scanned to identify any other relevant studies. We did not apply any language restrictions.

The literature was reviewed to select studies that met the following inclusion criteria: 1) randomized or quasi-randomized controlled trials. 2) Pregnant woman with a singleton pregnancy, whose fetus had been diagnosed with IUGR, according to reference values. 3) Studies that were compared of L-arginine or sildenafil citrate with placebo or no intervention. Studies were excluded if they did not meet all of these inclusion criteria.

Study selection: First, two authors (JC-C and PY-C) independently screened the search results to confirm citations with potential relevance. Second, we obtained the full text with potential relevance. Two authors (JC-C and PY-C) independently decided on trial inclusion. We resolved any differences in opinion by discussion.

Assessment of study quality: Both review authors independently assessed the quality of each trial using the criteria outlined in Cochrance Handbook for Systematic Reviews of Interventions. Study quality was judged by the design and execution of randomization, concealment of allocation, completeness of follow up and blinding.

Statistical calculations were performed using the computer programs STATA 12 statistical software (STATA corp, Tex). For dichotomous data, we presented results as risk ratios (RRs) with 95 % confidence intervals (95 % CIs). Data were combined for a meta-analysis to calculate a pooled estimate of a treatment effect for each outcome. For continuous data, we reported the standardized mean difference (SMD). Summary estimates of SMDs or RRs were obtained with a random effects model. An I^2^ statistic was used to assess heterogeneity between trials. Heterogeneity was determined by a random effects model. If substantial heterogeneity was found in updates (I^2^ more than 50 %), subgroup analyses for main outcomes were performed. Potential publication bias was assessed with Egger’s test. A two-sided *P* value less than 0.05 was regarded as significant difference for all analyses.

## Results

### L-arginine on IUGR

The literature search yielded 1871 articles, of which 57 full texts were reviewed; the process was shown in Additional file 1. Of these studies, 8 randomized controlled trials and one quasi-randomized controlled trial [8] met the inclusion criteria. The number of patients in each trial ranged from 41 to 108 and totaled 576. However, two studies did not specify the number of IUGRs during pregnancy and one study only indicated a ratio of IUGR [9]. Other studies specified IUGR after a fetus had been delivered. In these trials, the expected fetal body weight between the control/placebo groups and arginine treatment groups were similar by ultrasound when they entered the study. The gestational ages at the beginning of treatment and at delivery are listed in Table 1. One study diagnosed IUGR in the mid-trimester, IUGR in this study was related to severe IUGR [10].

**Table 1:** Characteristics of 9 studies eligible for inclusion in meta-analysis

Studies	ARG/PG (or control group)	Inclusion criteria of IUGR fetuses (Fetal weight)	Disease of pregnancy	Maternal age (wk, ARG/ PG)	Gestational age at entry (wk, ARG/ PG)	Daily dose and supplement type	Course of treatment
Sieroszewski et al. 2004 [14]	78/30	Below 10th percentile for GA	NA	27.1 ± 5.2/29.5 ± 7.4	29.5 ± 2.7/31.7 ± 3.6	3 g/d, oral	20 days
Xiao and Li 2005 [8]	30/36	Below 10th percentile for GA	NA	27.0 ± 3.6/27.4 ± 3.1	33.03 ± 2.59/33.06 ± 2.43	20 g/day, intravenous	7 days
Rytlewski et al. 2006 [15]	30/31	Below 10th percentile for GA	preeclampsia	29.3 ± 6.7/29.2 ± 5.9	29.3 ± 3.42/29.1 ± 3.41	3 g/d, oral	Until delivery
Dear et al. 2007 [9]	42/27	Below 10th percentile for GA	Gestational hypertension	28 ± 5/28 ± 4	31.09 ± 2.98/29.88 ± 3.22	3 g/d, oral	Until delivery
Ropacka et al. 2007 [12]	24/17	Below 10th percentile for GA	NA	28.7 ± 5.9/28.7 ± 3.8	31.2 ± 3.1/29.7 ± 3.4	3 g/d, oral	Until delivery
Zhang et al. 2007 [11]	35/33	Below 10th percentile for GA	Gestational hypertension	NA	NA	20 g/d, intravenous	14 days
Winer et al. 2009 [10]	21/22	Below 3rd percentile for GA	Preeclampsia,or other disease	28.2 ± 5.9/29.3 ± 4.2	28.0 ± 2.0/27.7 ± 2.1	14 g/d, oral	Until delivery
Shen and Hua 2011 [13]	30/30	Below 10th percentile for GA	NA	NA	NA	15 g/d, intravenous	NA
Singh et al. 2014 [16]	30/30	Below 10th percentile for GA	NA	25.47 ± 4.3/25.17 ± 4.3	33.13 ± 2.9/33.0 ± 2.3	3 g/d, oral	21 days

*ARG* L-Arginine treatment group, *PG* placebo group, *GA* gestational age, *NA* not available

We assessed the quality of each trial using the criteria outlined in Cochrance Handbook for Systematic Reviews of Interventions, the data were shown in Additional file 2. Overall, five studies were of good quality, and four were of uncertain quality.

**Fig. 1:**
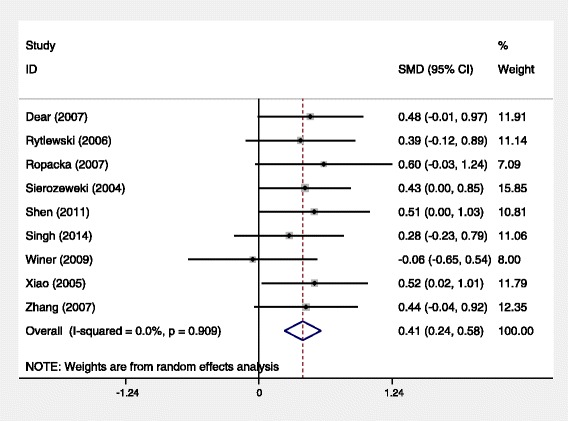
Random-effetcs meta-analysis comparing birth weight between control groups and L-aginine groups. The test for heterogeneity was not significant (*P* = 0.91),the standardized mean difference significantly favors controls (0.41, 95 % CI [0.24, 0.58], *P* < 0.0001)

The effects of L-arginine treatment on birth weight were shown in Fig. 1. Birth weight was the most important marker in evaluating the effect of L-arginine on IUGR. When data from all the studies were combined, the overall SMD was 0.41, (95 % CI [0.24, 0.58], *P* < 0.0001, heterogeneity: I^2^ = 0, *P* = 0.909). This indicated that the birth weight of IUGR fetuses significantly increased after arginine treatment.

**Fig. 2:**
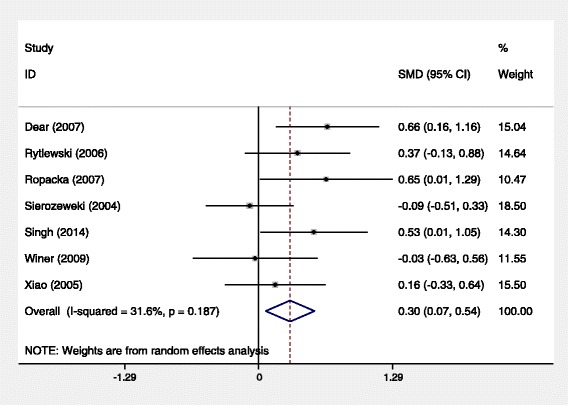
Random-effetcs meta-analysis comparing gestational age at labour between control groups and L-aginine groups. The test for heterogeneity was not significant (*P* = 0.19),the standardized mean difference significantly favors controls (0.30, 95 % CI [0.07,0.54], *P* = 0.012)

The effects of L-arginine therapy on gestational age at labor were shown in Fig. 2. Data on gestational age at labour were available for 7 trials. In these 7 trials, the fetuses were full-term births in one study [8] and preterm in the other studies. The combined SMD for gestational age at labor was 0.30, 95 % CI [0.07, 0.51], *P* = 0.012, heterogeneity: I^2^ = 31.6 %, *P* = 0.187. There was a significant difference between the two groups.

Table 2 summarizes other clinical outcomes (including the ratio of neonatal respiratory distress syndrome (NRDS) and intracranial hemorrhage (ICH), Apgar score at 1 and 5 min),and neonatal death. The ratio of NRDS was available for 4 trials [8, 9, 12, 16]. Compared with the control/placebo group, the ratio of NRDS in the treatment group was significantly reduced. The combined RR was 0.49, 95 % CI [0.29, 0.84], *P* = 0.009. Heterogeneity: I^2^ = 48 %, *P* for heterogeneity = 0.13. Two trials reported the ratio of ICH [8, 12]. Compared with the placebo group, the ratio of ICH in the treatment group was significantly reduced. The combined RR was 0.13, 95 % CI [0.03, 0.48], *P* = 0.002, heterogeneity: I^2^ = 0 %, *P* for heterogeneity = 0.90.

**Table 2:** Outcomes that the effect of L-arginine on neonate

	Included stuides (N)	ARG/PG (or control group)	Relative risk (95 % CI)	*P* value for overall effect	*P* value for heterogeneity
The ratio of NRDS	4	122/98	0.49 [0.29,0.84]	0.009	0.13
The ratio of ICH	2	66/44	0.13 [0.03,0.48]	0.002	0.90
Apgar score(>7) (1 min)	4	110/101	0.49 [0.23,1.03]	0.06	0.14
Apgar score(>7) (5 min)	4	101/94	0.28 [0.07,1.16]	0.08	0.05
Neonatal mortality	2	56/55	0.82 [0.19,3.66]	0.80	0.28

The effects of arginine treatment on the Apgar score (1 min, 5 min) in the individual studies are shown in Table 2. In the Dera et al. [9] study, the authors only reported the mean value and did not list the ratio of the related Apgar score. Therefore, the statistical data were not included in this study. The combined odds ratio for the Apgar score at 1 min (Apgar score more than 7) was RR 0.49, 95 % CI [0.23, 1.03], heterogeneity: I^2^ = 45 %, *P* for overall effect = 0.06. The combined RR for the Apgar score at 5 min was (Apgar score >7) 0.28, 95 % CI [007, 1.16] *P* = 0.08, heterogeneity: I^2^ = 67 %, *P* for heterogeneity = 0.05. Only two trial reported the ratio of neonatal mortality [15, 16], the data show that there wasn’t significant difference between the two groups (*P* = 0.80).

Table 3 summarizes the clinical outcomes from arginine in pregnant women. Maternal and umbilical blood serum levels of NO_2_/NO_3_ were available from 3 trials [8, 10, 11]. Compared to the control/placebo group, the serum NO_2_/NO_3_ levels in the umbilical and maternal blood of the treatment group was significantly different (*P* < 0.0001). A Doppler evaluation of umbilical arteries was available from 2 trials [11, 15]. Compared to the control group, the pulsatility index (PI) was significantly lower in the umbilical artery after three weeks of treatment (*P* = 0.02).

**Table 3:** Clinical outcomes that arginine effect on pregnant women: study include, odds risk, heterogeneity

	Studies	SMD (95 %CI)	*P* value
NO2/NO3 level in umbilical blood	3	0.66 [0.35,0.96]	<0.0001
NO2/NO3 level in maternal blood	3	0.70 [0.39,1.00]	<0.0001
Pulsatility indexes(PI) in umbilical artery	2	−1.98 [−3.65,−0.32]	0.02

There was no evidence of publication bias for the outcome of birth weight between two groups (Egger’s test *P* = 0.70).

The number of included studies and people on clinical outcomes (including the ratio of NRDS, ICH, Apgar score and neonatal mortality) was small. Therefore, there were insufficient data for reliable conclusions on the effects of L-arginine treatment on these clinical outcomes of IUGR fetuses.

### Effect of Sildenafil citrate on IUGR

Two trials compared Sildenafil citrate with either placebo or no intervention. One study [17] was a RCT, and the other [18] was a nonrandomized comparative study. The two trials both had positive outcomes (improvements in umbilical and middle cerebral arteries Doppler velocimetries). However, further analysis of the effect of sildenafil citrate on IUGR was not possible because the number of included trials was insufficient.

## Discussion

### Main findings

The aim of this meta-analysis was to assess the effectiveness of L-arginine and sildenafil citrate for treating IUGR. Ten small trials evaluated the effects of L-arginine or sildenafil citrate compared with a placebo or no intervention in women with an increased risk of IUGR. The combined results suggest that L-arginine increases fetal birth weight and prolong the gestational age at labor. These findings are in contrast with results from some review studies and individual trials that reported no benefit with these therapies [19, 20]. As only one trial compared sildenafil citrate with placebo on IUGR, no reliable conclusions about possible differential effects could be drawn.

Low birth weight is one of the most direct manifestations of IUGR, increased birth weight means the reduced number of IUGR fetuses. In our analysis, L-arginine significantly increased fetal birth weight (9 trials, SMD 0.41, 95 % CI [0.24, 0.58], *P* < 0.0001), and the arginine treatment in IUGR was effective in 8 trials. Only one study by Winer et al. reported negative results. However, the included patients were different between Winer et al’ study and other 8 trials, the IUGR in Winer et al’ study were severe IUGR, the infants also were very preterm infants (gestational age < 32 weeks). The concentration of L-arginine in pregnant women varied [21, 22]. A cohort study by Neri I et al. [23] evaluated pregnant women with mild or moderate growth-restricted fetuses and pregnant women with severe growth-restricted fetuses. After these patients were given L-arginine, the birth weight in the former group was significantly higher than in the latter group (birth weight was 2061 g vs 1608 g). Different patients included may lead to different outcomes in our meta-analysis. Regardless, more RCTs are needed to address this possibility.

As we mentioned above, IUGR is associated with increased risk of premature birth; prolonged latency (duration of pregnancy) results in a reduced rate of preterm birth. Prematurity is usually accompanied by adverse neonatal outcomes, such as NRDS, ICH, and low Apgar score. At the same time, the weight of fetus increases fastest during the third trimester of pregnancy, and prolonged latency also may increase birth weight. Some studies also showed IUGR was increased with prematurity (gestational age at labor 26 weeks = 8.9 %, 27 weeks = 7.7 %, 28 weeks = 9.8 %) [24]. In our study, our results show that L-arginine can prolonged gestational age in our study, it don‘t significantly reduce the rates of neonate mortality, and significantly increased the Apgar score (*P* > 0.05). These results are puzzling. We speculated that the included trials that L-arginine on major clinical outcomes were too few to reach consistent conclusions, so further studies are needed.

When L-arginine is taken orally, 40 % is degraded by the small intestine and metabolized by arginase in the liver. Thus, poor arginine availability in the blood may limit the efficacy of this amino acid [25, 26]. However, almost all studies in our study have positive results, though the daily doses and courses of treatment were different. The determination of the optimal duration of L-arginine therapy requires further research. In the oral groups, L-arginine was administered until delivery; in the intravenous group, it was given for 7 days, 14 days or 20 days. Rytlewski K et al. [15] demonstrated a significant increase in estimated fetal body weight after the first two weeks of treatment in their L-arginine group compared to the placebo group. However, the results from other trials were different [27, 28]. Therefore, more studies are necessary to clarify these results.

Some authors have reported some side effects of L-arginine in other studies, such as diarrhea, disturbance of acid–base balance, etc [29–31]. However, none of the 9 trials in this study described any L-arginine side effects. Additionally, no teratogenic or lethal effects were reported. The number of included studies in our meta-analysis is small, so it is imperative that future studies be designed to address these side effects.

Animals studies had shown that L-arginine was beneficial to IUGR and the other mechanisms of effect of arginine on fetus growth include: 1) Creatine. L-arginine can produce creatine that is one of materials which make form muscle. Arginine also can stimulate skeletal muscle protein synthesis [32, 33]. 2) Growth hormone. L-arginine is also found to improve growth hormone releasing hormone secretion, and as a consequence increase in plasmatic growth hormone influencing somatic growth [34]. 3) L-arginine was reported to promote polyamine synthesis that can enhance placental growth and development [35]. 4) Arginine can stimulate insulin secretion, insulin is a major anabolic hormone in the fetus [23].

As a vasodilator, sildenafil citrate can reduce vasoconstriction and improve relaxation of IUGR myometrial small arteries, which may improve oxygen and nutritional supply to the fetus [36, 37]. Some experimental studies have supported sildenafil citrate as a potential candidate for the treatment of IUGR in both animals and human [38–41]. However, there is limited clinical information proving the efficacy of sildenafil in treating IUGR. Therefore, more RCTs are needed to be designed to determine the effect of sildenafil citrate on IUGR.

There were several limitations in our meta-analysis. First, the number of included trials was very small, and only one RCT that comparing sildenafil citrate with placebo was included. Second, the sample sizes of these trials were quite small. Third, the dose and the route of L-arginine administration varied, potentially leading to uncertainty biases in the final result of the meta-analysis.

## Conclusions

According to our meta-analysis, L-arginine supplementation is superior to placebo in increasing birth weight and prolonging gestational age at labor of IUGR fetuses, but the data for L-arginine on other clinical outcomes (like the ratio of NRDS,ICH, Apgar score and neonatal mortality) were insufficient. At this time, there is insufficient evidence to draw conclusions about L-arginine supplementation in IUGR. Large-scale, multicenter RCTs are needed to determine whether arginine supplementation has a beneficial role in IUGR disorders. Only one trial compared sildenafil citrate with placebo, There is limited information on the efficacy of sildenafil citrate for treating IUGR.

## Abbreviations

ICH, intracranial hemorrhage; IUGR, intrauterine growth restriction; NO, nitric oxide; NRDS, neonatal respiratory distress syndrome; RCTs, randomized controlled trials; RR, risk ratios; SGA, small for gestational age; SMD, standardized mean difference

## Additional files

Additional file 1: Identification process for eligible studies. (DOC 35 kb) Additional file 2: Quality of enrolled RCTs [8–16]. (DOC 40 kb)
