# Supplementary material for: Effect of L-arginine and sildenafil citrate on intrauterine growth restriction fetuses: a meta-analysis
Source: BMC Pregnancy Childbirth. 2016 Aug 16;16:225. doi: 10.1186/s12884-016-1009-6 (PMC4986189; doi:10.1186/s12884-016-1009-6)
Supplement: Additional file 1: — Identification process for eligible studies. (DOC 35 kb) [file 12884_2016_1009_MOESM1_ESM.doc]

**Additional file 1: Identification process for eligible studies**

Records identified through database searching (n = 1823)

Additional records identified through other sources (n = 48)

Records after duplicates removed (n =945)

Records screened (n = 89)

Records excluded (n = 856)

Full-text articles assessed for eligibility (n =57)

Studies included in qualitative synthesis (n = 11)

Studies included in quantitative synthesis (meta-analysis) (n =10)

L-arginine 9

sildenafil citrate 1

Excluded because did not report

Sufficient information to include in

the meta-analysis.

*n* = 1

**Identification**

**Screening**

46 studies Excluded

28Fetuses not IUGR fetuses (animals , low birth weight or normal fetuses)

6 were cohort study or retrospective study, not RCTs

7 neonatal outcomes without birth weight or ratio of IUGR

2 fetuses didn’t be delivered

3 medicine was mix acid amino

**Eligibility**

**Included**
