# Supplementary material for: Effect of L-arginine and sildenafil citrate on intrauterine growth restriction fetuses: a meta-analysis
Source: BMC Pregnancy Childbirth. 2016 Aug 16;16:225. doi: 10.1186/s12884-016-1009-6 (PMC4986189; doi:10.1186/s12884-016-1009-6)
Supplement: Additional file 2: — Quality of enrolled RCTs [8–16]. (DOC 40 kb) [file 12884_2016_1009_MOESM2_ESM.doc]

**Additional file 2:** Quality of enrolled RCTs.

| Studies | Randomization | Blinding | Outcomes | Notes | Risk of bias | |
| --- | --- | --- | --- | --- | --- | --- |
|  |  |  |  |  | Allocation concealment? | Description |
| Sieroszewski et al, 2004 [14] | Yes | Not clear | Women: 21 patients discontinued | Data on side-effects not reported for both groups | Yes | A-adequate |
| Xiao and Li, 2005 [8] | quasi-randomized | No | Women: No subject lost. NO levels in plasma. | Data on side-effects not reported for both groups | Not-clear | B-Unclear |
| Rytlewski et al, 2006 [15] | Yes | Yes | Women: 22 patients discontinued.  Baby: Apgar score; neonatal mortality. | Data on side-effects not reported for both groups | Yes | A-adequate |
| Dera et al,  2007 [9] | Yes | Yes | Women: No subject lost; abruption.  Baby: Apgar score; complication; neonatal mortality; resuscitations  . | Data on side-effects not reported for both groups | YES | A-adequate |
| Ropacka et al,  2007 [12] | Yes | Yes | Women: No subject lost ; abruption.  Baby: Apgar score; complication; neonatal mortality; resuscitations. | Data on side-effects not reported for both groups | YES | A-adequate |
| Zhang et al, 2007 [11] | Yes | Not clear | Women: the number of discontinued women was not clear; NO levels in plasma. | Data on side-effects not reported for both groups | Not clear | B-Unclear |
| Winer et al,  2009 [10] | Yes | Yes | Women: 3 patients discontinued; abruption; complication. Baby: Apgar score and CRIB score. | Data on side-effects not reported for both groups | Yes | A-adequate |
| Shen and Hua, 2011 [13] | Yes | Not clear | Women: the number of discontinued women was not clear; NO levels in plasma. | Data on side-effects not reported for both groups | Not clear | B-Unclear |
| Singh et al, 2015 [16] | YES | Not clear | Women: No subject lost; abruption; NO levels in plasma.  Baby: Apgar score; complication; neonatal mortality; resuscitations. | Data on side-effects not reported for both groups | Not clear | B-Unclear |
